# Spontaneous Psoas Hematoma Following Resistance Training in an Elderly Patient on Apixaban: A Case Report

**DOI:** 10.7759/cureus.87748

**Published:** 2025-07-11

**Authors:** Violeta Foss, Taner B Celebi, Rafeh Waheed, Herman Dhillon

**Affiliations:** 1 Family Medicine, Northwell Health, Plainview, USA; 2 Radiology, Northwell Health, Manhasset, USA

**Keywords:** atrial fibrillation (afib), : direct oral anticoagulants (doac), exercise training, iliopsoas hematoma, oral anticoagulation

## Abstract

Regular physical activity, including resistance training, is strongly recommended for older adults to preserve muscle mass, balance, and functional independence. However, the increasing use of direct oral anticoagulants (DOACs), such as apixaban, in this population introduces important clinical considerations, particularly the risk of spontaneous bleeding in those participating in physical activity. Various scoring systems are used to create better judgement calls when initiating an anticoagulant, however initiating the anticoagulant is up to the discretion of the physician. We present the case of an 80-year-old male with multiple comorbidities, including atrial fibrillation managed on apixaban for anticoagulation, who was diagnosed with a spontaneous psoas hematoma during hospitalization for pneumonia. This case highlights the importance of a risk-aware approach to physical activity counseling in older adults on anticoagulation therapy. Clinicians must maintain a high index of suspicion and provide tailored exercise guidance to minimize injury risk. As the older population and use of DOACs continue to rise, increased awareness and surveillance are essential to promote safe physical activity in this vulnerable group.

## Introduction

Physical activity remains a cornerstone of preventive care, particularly in the aging population. The American Heart Association recommends that adults engage in at least 150 minutes of moderate or 75 minutes of vigorous aerobic activity per week, along with two days of moderate- to high-intensity resistance training [1]. These recommendations are critical in older adults, who experience age-related muscle mass decline starting at age 30, with an accelerated reduction after age 60. Resistance training is an exercise modality that can help mitigate the decline in muscle mass, which is associated with poor balance, falls, as well as impaired functional capacity and quality of life. 

Simultaneously, there is growing use of direct oral anticoagulants (DOACs), particularly in older adults [2]. Apixaban, a factor Xa inhibitor, is frequently prescribed for stroke prevention in patients with atrial fibrillation. More than 10 million Americans are estimated to have atrial fibrillation, and those aged ≥65 years comprise a significant portion of this population [3]. Remarkably, individuals over 65 account for more than one-third of all ambulatory care visits in the United States [4]. 

While apixaban is considered safer than warfarin based on its side effect profile and risk factors, it still carries a risk of spontaneous or trauma-induced bleeding. Spontaneous muscle hematomas, though uncommon, have been reported in patients on DOACs [5,6]. Physical stress from resistance training may further increase this risk. Therefore, clinicians must incorporate anticoagulation status into exercise counseling to prevent serious complications, such as internal hemorrhage. 

## Case presentation

An 80-year-old male with a history of paroxysmal atrial fibrillation, chronic diastolic heart failure, hypertension, hyperlipidemia, and type 2 diabetes mellitus presented with acute hypoxic respiratory failure secondary to pneumonia in the context of chronic obstructive pulmonary disease (COPD). 

**Figure 1:**
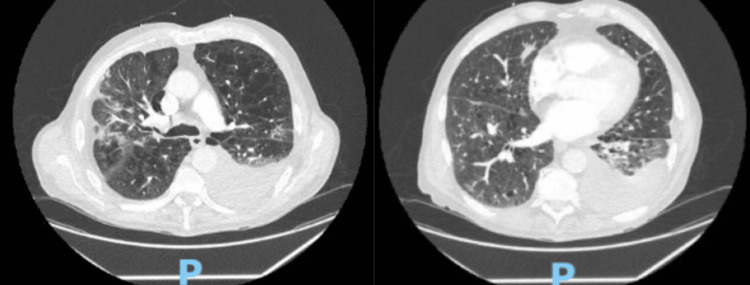
CT angiography of the Chest CT angiography of the chest showing severe emphysema, as well as bilateral patchy ground-glass opacities. Axial images demonstrate the ground-glass opacities most prominent in the right upper and left lower lobes. Pleural effusion noted on left side.

At admission, the patient met sepsis criteria with a heart rate of 96 beats per minute and a respiratory rate of 26 breaths per minute while on 5 L/min oxygen via nasal cannula. Chest computed tomography (CT) revealed patchy ground-glass opacities in the right upper and left lower lobes as illustrated in Figure 1. He was started on ceftriaxone and azithromycin for community-acquired pneumonia, with azithromycin also selected for its anti-inflammatory properties. A short course of oral prednisone (10 mg twice daily for two days) was administered. Pulmonology was consulted to assist with treatment planning. 

The patient’s home apixaban (5 mg twice daily) was continued for history of atrial fibrillation requiring full anticoagulation. On hospital day three, he reported left inguinal pain, which had started prior to admission. He denied trauma but disclosed a home exercise routine involving daily squats and resistance training where he recently achieved a personal best in lifting a heavier weight. 

**Figure 2:**
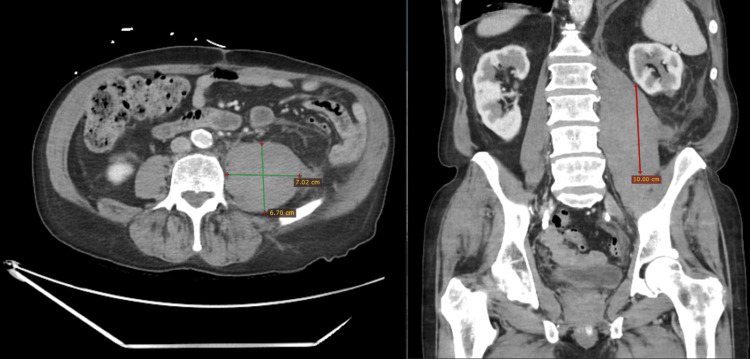
Contrast-enhanced CT of the abdomen and pelvis showing the largest cross-section of a left psoas intramuscular hematoma. Axial and coronal views demonstrate the hematoma measuring approximately 6.0 × 7.0 × 10.0 cm within the retroperitoneal space.

Physical examination revealed mild tenderness in the left inguinal area without ecchymosis, edema, or skin changes. The pain worsened with hip flexion. Genitourinary and integumentary examinations were unremarkable. Given the patient’s history of nephrolithiasis, a non-contrast CT abdomen and pelvis was performed, showing enlargement of the left psoas muscle. A follow-up CT with intravenous contrast confirmed an intramuscular hematoma measuring 6.0 × 7.0 × 10.0 cm (Figure 2). Both hemoglobin and platelet count decreased from admission to day three of hospitalization (Table 1).

**Table 1:** Hemoglobin and platelet count from day of admission Hemoglobin (Hb) and platelet count (Plt) on day of admission, day three, and day five of hospitalization with their associated normal reference values.

Labs	On Admission	Day 3	Day 5	Ref. Range
Hb	12.2 g/dL	10.2 g/dL	10.6 g/dL	13.0-17.0 (g/dL)
Plt	320 K/uL	239 K/uL	---	150-400 (K/uL)

Apixaban was held due to evidence of active bleeding. General Surgery and Cardiology were consulted and recommended conservative management and close monitoring. No surgical intervention was required during the hospital stay. The patient’s respiratory symptoms improved following five days of ceftriaxone, three days of azithromycin, and a two-day prednisone course. Blood cultures remained negative throughout the hospital stay. 

Apixaban resumed after five days when the hemoglobin stabilized at 10.6 g/dL. The patient did not require blood transfusions during his hospitalization as the hemoglobin never dropped below 8.0 g/dL. He was discharged with instructions to avoid strenuous activity, particularly squats or heavy resistance training. Follow-up with his primary care physician within two to three days was advised for repeat CBC and continued outpatient surveillance, including possible follow-up imaging. 

## Discussion

This case underscores the complex interplay between exercise promotion, anticoagulation therapy, and the physiological vulnerabilities of the aging population. The patient, an active 80-year-old male on apixaban for atrial fibrillation, was found to have a spontaneous psoas hematoma, likely precipitated by routine resistance training in the context of anticoagulation and was only found because he arrived at the hospital and received treatment for his pneumonia. Hematomas have been illustrated in the AMPLIFY and AMPLIFY-EXT study to occur in 1.3% -2.0% of cases when being treated for deep vein thrombosis (DVT) and pulmonary embolism (PE), though size and location have always been irregular [7,8]. This presentation highlights the crucial need for nuanced and specific exercise counseling tailored to older adults with chronic medical conditions. 

DOACs, such as apixaban, have become the standard of care in preventing thromboembolic events in patients with atrial fibrillation, largely because of their favorable safety profile compared to warfarin [7,8]. However, despite a reduced risk of major bleeding events, DOACs still have risks of complications. Spontaneous muscle hematomas, specifically psoas and rectus sheath hematomas, have been increasingly reported, especially in elderly patients or those engaging in physical activity [5,6]. Bouget et al. illustrated in a multicenter cohort study that major muscle hematomas associated with antithrombotic agents occurred in 13 out of 375. In the same study, more specifically, rectus sheath hematomas occurred in 71 cases and iliopsoas hematomas accounted for 45 cases [9]. This patient's case fits this pattern, with symptom onset following exercise and in the absence of trauma or invasive procedures. 

The pathophysiology behind this type of hematoma involves microvascular trauma within muscle compartments [10]. In anticoagulated individuals, even minor muscle strain from resistance training, especially exercises involving eccentric contraction such as squats, may lead to vascular injury and bleeding. The psoas muscle, being deep and subject to significant mechanical stress during lower body exercises, is particularly vulnerable. Compounding this is the fact that older adults often have fragile vasculature and decreased muscle elasticity, further increasing their risk. Unfortunately, the exact frequency of these types of hematomas in patients is not well documented in the current literature and may be a path to further explore to best educate patients who appreciate being active and exercise to prevent this type of pathology. 

Clinically, spontaneous psoas hematomas can present insidiously, with pain that may mimic other common geriatric complaints such as renal colic, musculoskeletal pain, or even neuropathy [10]. In this case, the initial presentation was vague inguinal discomfort without external signs of bleeding. The lack of visible ecchymosis and the normal genitourinary and integumentary exam delayed initial suspicion. It was only through careful history, including acknowledgment of the patient's exercise routine, and appropriate imaging that the diagnosis was confirmed. This reinforces the importance of a high index of suspicion for bleeding complications in anticoagulated patients, even when classic signs are absent. 

There can be different contributing factors in the case of retroperitoneal hematoma with DOAC use. This patient’s primary concern for hospitalization was pneumonia and a cough in the setting of underlying COPD. Forceful or repetitive coughing increases intrathoracic and intra-abdominal pressure. As a result, in an older patient on apixaban, even a minor blood vessel rupture or muscle layer tear may cause a hematoma large enough to be symptomatic. While pneumonia may have been a potential culprit for initiating the psoas injury in his patient’s case, most reported cases of hematomas due to cough on DOACs are associated with the rectus sheath [10-12]. Additionally, the patient’s cough had markedly improved by the time he began experiencing inguinal pain, likely due to the psoas hematoma enlarging. As he continued to improve clinically from a respiratory standpoint, the patient made a concerted effort to ambulate frequently during the hospitalization. He felt that his physical activity had been insufficient during the peak of his respiratory symptoms. The increase in ambulation coinciding with inguinal pain onset, along with the hematoma location in the psoas muscle rather than the rectus sheath, suggests movement as the more likely precipitating factor in this case. 

Management of such hematomas is typically conservative, involving discontinuation or temporary withholding of the anticoagulant, supportive care, and monitoring for hemodynamic stability. Surgical intervention is rare and usually reserved for expanding hematomas or those causing neurovascular compromise [9,11]. In this case, coordination among Internal Medicine, Cardiology, and General Surgery allowed for effective interdisciplinary care. The patient’s apixaban was held with serial monitoring of hemoglobin, and no procedural intervention was necessary. 

This case also raises a critical point for primary care providers who counsel older adults on exercise. While resistance training remains a cornerstone of healthy aging, preserving muscle mass, improving balance, and enhancing metabolic health, it must be prescribed with individualized caution in anticoagulated patients. Providers should assess bleeding risk using validated tools (e.g., HAS-BLED score), evaluate exercise intensity, and counsel patients on signs of internal bleeding [12]. Emphasis should be placed on low-impact, controlled movements, with avoidance of strenuous or high-resistance lower-body activities, unless cleared by a physician. 

## Conclusions

Structured physical activity, particularly moderate- to high-intensity resistance training, can help preserve muscle mass, especially in older adults. While exercise is essential for healthy aging, its benefits must be carefully balanced against the risks associated with anticoagulant therapy. DOACs, such as apixaban, directly inhibit certain factors in the blood clotting cascade and offer significant benefits for anticoagulation in a large portion of the older adult population. However, the class of medications also carries the inherent risk of severe bleeding. The blood loss resulting from DOAC use can range from minor mucosal bleeds to potentially life-threatening internal hemorrhage. Given the possibility of these grave consequences associated with DOACs, it is critically important to thoughtfully consider the implications of moderate to high-intensity exercise and provide targeted guidance for patients who take apixaban. This case illustrates the importance of individualized exercise counseling for patients on DOACs. Heightened clinical vigilance, patient education, and tailored physical activity guidelines are key to preventing adverse outcomes such as spontaneous muscle hematomas in this growing patient population. Proactive primary care is paramount for prevention, as well as risk mitigation, of such complications. Family medicine physicians are the cornerstone of achieving this goal. 
